# Ultrafast time-resolved x-ray absorption spectroscopy of ionized urea and its dimer through *ab initio* nonadiabatic dynamics

**DOI:** 10.1063/4.0000076

**Published:** 2021-05-12

**Authors:** Yashoj Shakya, Ludger Inhester, Caroline Arnold, Ralph Welsch, Robin Santra

**Affiliations:** 1 Center for Free-Electron Laser Science CFEL, Deutsches Elektronen-Synchrotron DESY, Notkestr. 85, 22607 Hamburg, Germany; 2 Department of Physics, Universität Hamburg, Notkestr. 9-11, 22607 Hamburg, Germany; 3 Hamburg Centre for Ultrafast Imaging, Luruper Chaussee 149, 22761 Hamburg, Germany

## Abstract

Investigating the early dynamics of chemical systems following ionization is essential for our understanding of radiation damage. However, experimental as well as theoretical investigations are very challenging due to the complex nature of these processes. Time-resolved x-ray absorption spectroscopy on a femtosecond timescale, in combination with appropriate simulations, is able to provide crucial insights into the ultrafast processes that occur upon ionization due to its element-specific probing nature. In this theoretical study, we investigate the ultrafast dynamics of valence-ionized states of urea and its dimer employing Tully's fewest switches surface hopping approach using Koopmans' theorem to describe the ionized system. We demonstrate that following valence ionization through a pump pulse, the time-resolved x-ray absorption spectra at the carbon, nitrogen, and oxygen *K*-edges reveal rich insights into the dynamics. Excited states of the ionized system give rise to time-delayed blueshifts in the x-ray absorption spectra as a result of electronic relaxation dynamics through nonadiabatic transitions. Moreover, our statistical analysis reveals specific structural dynamics in the molecule that induce time-dependent changes in the spectra. For the urea monomer, we elucidate the possibility to trace effects of specific molecular vibrations in the time-resolved x-ray absorption spectra. For the urea dimer, where ionization triggers a proton transfer reaction, we show how the x-ray absorption spectra can reveal specific details on the progress of proton transfer.

## I. INTRODUCTION

The response of biological matter to ionizing radiation is of fundamental interest to many fields, ranging from radiation oncology,^1^ x-ray diffraction imaging,^2^ and human space flight.^3^ The dynamics in a molecule that are triggered by the formation of deep valence holes, i.e., by ionization beyond the highest occupied molecular orbital (HOMO), are of particular relevance for highly energetic ionizing radiation. Deep valence holes can be caused either by the direct interaction with extreme ultraviolet light or by secondary ionizations, e.g., by photoelectrons or Auger electrons following core-level ionization.^4–7^ Upon ionization, the molecule undergoes chemical dynamics that involve the conversion of the electronic excitation energy to vibrational energy through nonadiabatic couplings of electronic potential energy surfaces (PES).

The advent of x-ray free-electron lasers (XFEL) and the recent progress in high harmonic generation (HHG) technology offer new routes to investigate the ultrafast dynamics triggered by such ionization events in molecules. In particular, a promising tool to investigate ultrafast structural dynamics on a femtosecond timescale is time-resolved x-ray absorption spectroscopy (TRXAS), where the ultrafast dynamical changes initiated by an ionizing or exciting light pulse are probed with a delayed, ultra-short x-ray pulse. Complementary to the wide range of experimental methods that have been used to study the femtosecond dynamics of molecules,^8,9^ TRXAS has the advantage that the probe acts locally on a specific atomic site in the molecule since the dominant interactions occur with the core orbitals in the molecule. Different atoms with distinct inner-shell binding energies thus provide separate time-resolved absorption signals that cover different local aspects of the dynamics of the molecule.

The capabilities of TRXAS in probing structural and electronic dynamics on a femtosecond timescale have been demonstrated in a number of experiments at large XFEL facilities^10–15^ and in laboratory setups employing HHG.^16–18^ Interpreting the TRXAS signals is, however, usually not trivial and often relies crucially on theoretical modeling. In this context, a number of theoretical works have provided valuable insight into the dynamics following ionization or excitation of a sample molecule and how they can be revealed from TRXAS.^19–24^

Depending on the initial excitation level, the triggered dynamics can involve a large number of different PES.^19^ Hence, with increasing system size, simulating the dynamics and calculating the resulting TRXAS can become rather computationally involved, thus posing a strong challenge for the theoretical modeling. A relatively affordable, yet reasonably accurate, approach is to employ the fewest switches surface hopping method^25^ in combination with PES provided by Koopmans' theorem.^15,19,23,26,27^

In this work, we present a computational study on the TRXAS of urea and its dimer. Due to its use as a fertilizer, urea is a highly relevant molecule for industry and agriculture. Its first synthesis by Wöhler^28^ is often considered a foundational milestone in organic chemistry.^29^ As an amide with a planar structure it has similarities with proteins and thus can be seen as a simplified building block of a biological molecule. Moreover, the urea dimer is linked via hydrogen bonds whose specific role in TRXAS has not yet been studied. We have performed on-the-fly *ab initio* nonadiabatic dynamics following instantaneous HOMO and deeper valence ionization of urea and its dimer and calculated the TRXAS at the carbon (C), nitrogen (N), and oxygen (O) *K*-edges. Using these results, we demonstrate how statistical analysis can provide a clear interpretation of features in the TRXAS with respect to structural dynamics in the molecule. Furthermore, we reveal pronounced fingerprints of nonadiabatic relaxation and proton transfer reaction in the TRXAS.

The article is structured as follows: first, we briefly explain the computational methodology in Sec. II. In Sec. III, we present and discuss our results for the urea monomer and dimer. Final conclusions are drawn in Sec. IV.

## II. COMPUTATIONAL METHOD

We have performed on-the-fly *ab initio* molecular dynamics on multiple coupled electronic PES employing the methodology used in Refs. 15 and 23. Electronic structure calculations at a particular nuclear geometry have been performed using the XMOLECULE package (rev. 3700)^30,31^ at the Hartree–Fock (HF) level of theory with the 6-31+G^*^ basis set.^32–35^ To test for convergence, we also performed calculations with the smaller 6-31G basis set which gives qualitatively similar results. The ionized system has been described by Koopmans' theorem, which gives the energy of a state with a hole in the ith orbital as $E_{i}=E_{\text{HF}}-\epsilon_{i}$, where *ϵ_i_* is the energy of the ith orbital and $E_{\text{HF}}$ is the HF ground-state energy of the neutral molecule. This approach provides a computationally efficient, yet reasonable, description of the valence shell ionized system and was previously used to study the dynamics of other ionized systems.^15,19,23,26,27^ Although the accuracy is certainly limited, the employed scheme has the potential to be applied also to larger molecules, since it essentially only involves HF calculations. Due to its simplicity, it can also straightforwardly be applied to ionization of deeply bound valence orbitals where the ensuing relaxation dynamics involve a large number of different PES.^19^

In our calculations, the gradients of the PES and the nonadiabatic coupling vectors between the different ionized states have been obtained by solving the coupled perturbed Hartree–Fock equations in XMOLECULE.^23,36–38^

The molecular dynamics simulation on the ionized states has been performed using Tully's fewest switches surface hopping method.^25^ In this quantum-classical approach, the nuclei are treated classically on a single PES. At each time step, there is a probability to hop to another surface based on the coherent evolution of an electronic wavefunction. The propagation of the quantum electronic wave packet is done by solving the time-dependent Schrödinger equation simultaneously along with the classical propagation of the nuclear geometry.

Initial positions and momenta have been obtained by sampling the Wigner distribution of the ground vibrational state of the neutral molecule, employing the harmonic approximation. An ensemble of 100 samples has been created and then propagated starting in a selected valence-ionized electronic state for a total time of 300 fs using a simulation time step of 0.5 fs.

Along each trajectory, we have calculated the time-dependent x-ray absorption spectra. Given the element specificity of core orbitals, one can probe the dynamics of the molecule through different inner-shell edges in the x-ray absorption spectra, reflecting different local environments in the molecule. We have considered here the C, N, and O *K*-edges, whose binding energies are around 289 eV, 399 eV, and 535 eV, respectively.^39^ More specifically, we have addressed the energetically lowest absorption resonance that can be found below the ionization threshold and can be associated with the refilling of the previously created valence hole from the respective core orbital. Such an absorption resonance is thus only present in the ionized molecule, which in a prospective pump-probe experiment has the advantage that the signal from pumped molecules can be distinguished from the background signal of nonpumped, neutral molecules.

For an electron in the core orbital $\phi_{i}$ with orbital energy *ϵ_i_* being excited to the valence orbital vacancy $\phi_{f}$ with orbital energy *ϵ_f_*, the cross section has been calculated using^40^

$$\sigma (\omega )=\frac{4}{3}\pi^{2}\omega \alpha \;\delta (\epsilon_{f}-\epsilon_{i}-\omega )\;|\langle \phi_{f}|\hat{d}|\phi_{i}\rangle {|}^{2},$$ (1)

where *ω* is the photon energy of the x-ray probe pulse, *α* is the fine structure constant and $\hat{d}$ is the dipole vector, which in Eq. (1) is averaged over the three spatial dimensions. The quantities in Eq. (1) are given in atomic units (a.u.). For the function δ(E−ω), we have employed a finite-width line profile given by a Lorentzian function with a width of 100 meV to account for the natural linewidth of the core-ionized state.

We note that the energetic positions of the absorption resonances, here calculated by $\epsilon_{f}-\epsilon_{i}$, are several eVs off from realistic values. This is mostly due to orbital relaxation effects accompanying core-shell ionization that are not taken into account in the Koopmanns' approach. To correct for this effect, we have down-shifted the calculated absorption spectra by 21 eV, 25 eV, and 24 eV for the C, N, and O *K*-edges, respectively. These shifts have been estimated from the difference between the experimental K-edge ionization potentials and the calculated orbital binding energies.^39^

## III. RESULTS

### A. Urea

**FIG. 1.**
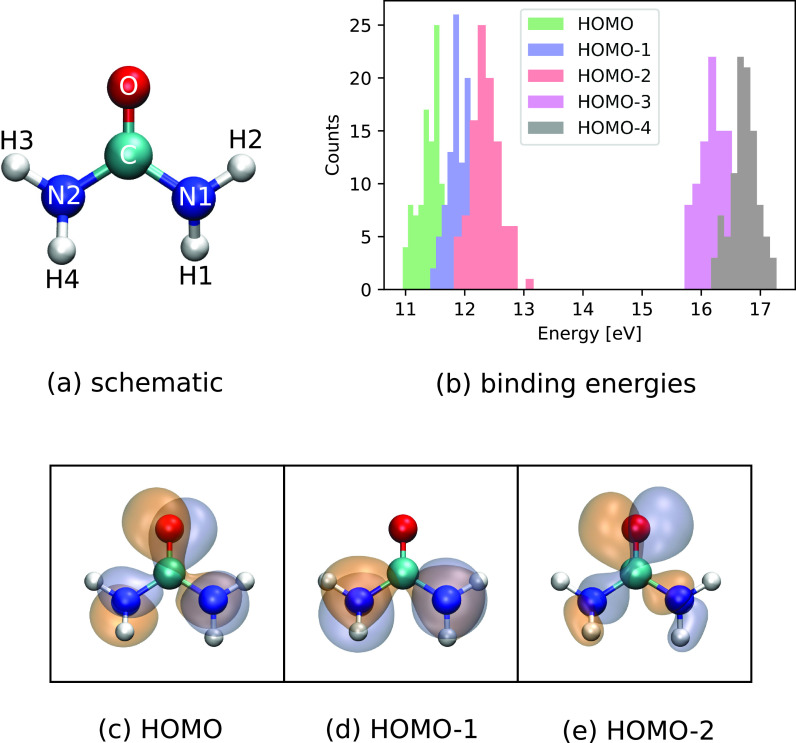
(a) Schematic of urea. (b) Histogram of the calculated molecular orbital binding energies for HOMO to HOMO-4 for the ground-state Wigner distribution of neutral urea. (c)–(e) HOMO, HOMO-1, and HOMO-2 at equilibrium geometry, visualized using VMD.^42^

For the urea molecule [schematic shown in Fig. 1(a)], we have considered initial ionization in the HOMO, HOMO-1, and HOMO-2. The orbitals are depicted in Figs. 1(c)–1(e). Figure 1(b) shows the corresponding binding energy histogram for the molecular geometries in the considered ground-state Wigner distribution. As can be seen, HOMO, HOMO-1, and HOMO-2 lie relatively close to each other in binding energy within the range between 11 eV and 13 eV. The computed binding energies are somewhat higher than the values from experimental data reporting 10.28 eV and 10.78 eV for the first two ionization potentials.^41^ As the binding energies overlap, one can expect that ionization of HOMO-1 or HOMO-2 leads to a rapid internal conversion via nonadiabatic dynamics toward the electronic ground state with a hole in HOMO. Additionally, these hole states are below the double ionization potential (∼30 eV as estimated from a ΔSCF calculation at the equilibrium geometry) and therefore do not undergo autoionization processes.

#### 1. TRXAS

**FIG. 2.**
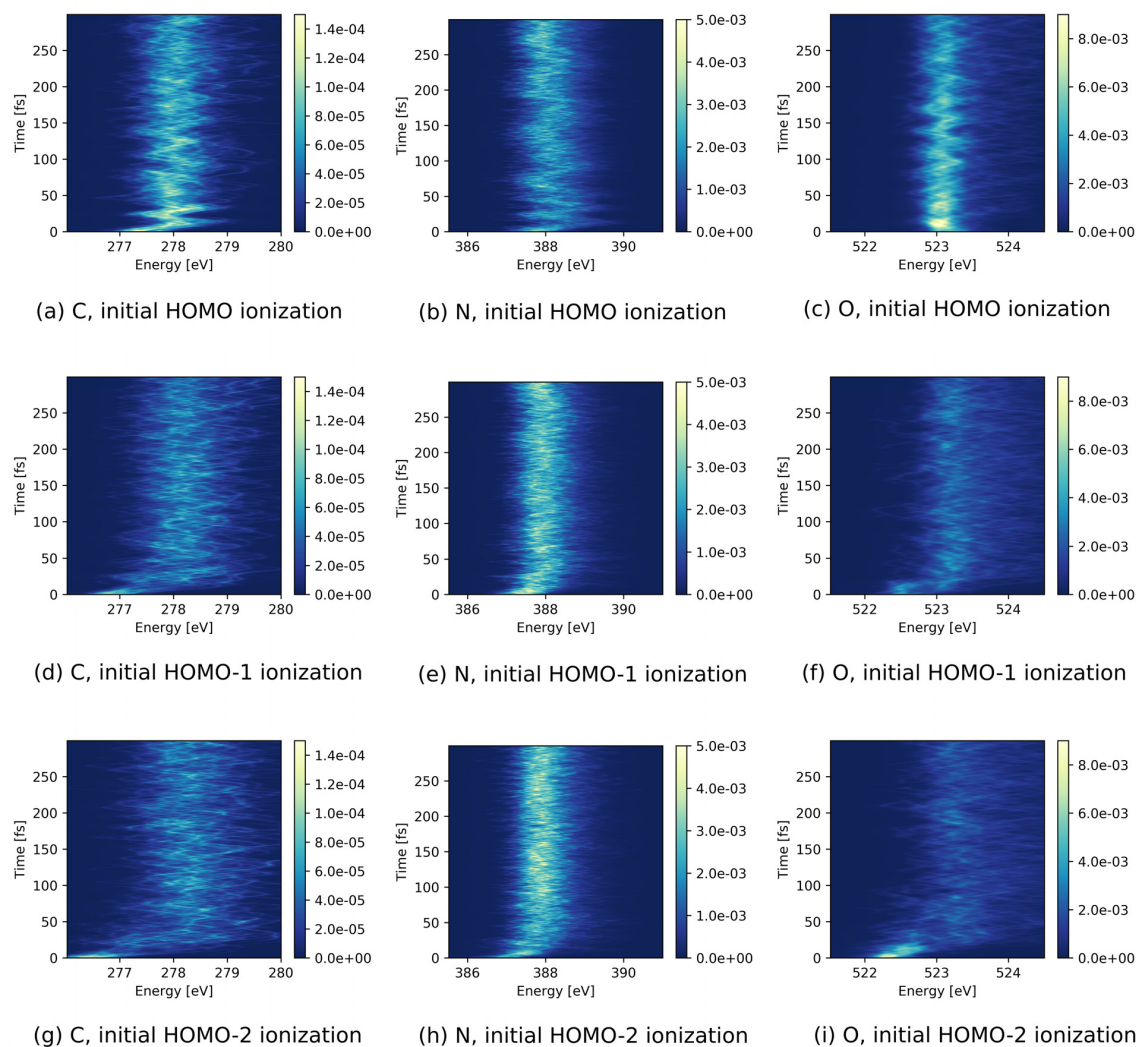
TRXAS (given by cross section in a.u.) of urea after initial ionization of (a)–(c) HOMO, (d)–(f) HOMO-1, and (g)–(i) HOMO-2 at the lowest (left) C, (middle) N, and (right) O *K*-edge absorption resonances.

Figure 2 shows the evolution of the three calculated x-ray absorption spectra after removing an electron from HOMO, HOMO-1, and HOMO-2, respectively. Compared to the N and O *K*-edges, the absorption at the C *K*-edge is considerably weaker, owing to the fact that the three considered valence orbitals only have a small amplitude on the C atom (see Fig. 1).

The three element-selective absorption spectra for an initial hole in HOMO [Figs. 2(a)–2(c)] exhibit oscillations in energy, in particular for the C and N *K*-edge absorption resonances during the first 50 fs. We attribute these oscillations to vibrations initiated by the ionization. Such oscillations are not observed for deeper hole states [Figs. 2(d)–2(i)], where nonadiabatic relaxation effects and higher vibrational energy apparently lead to a rapid decay of vibrational coherence. Along the same line, one can understand that the absorption resonances are considerably broader after initial ionization in HOMO-1 or HOMO-2 as compared to initial ionization in HOMO. Moreover, the spectra for the deeper hole states exhibit a blueshift of 1–2 eV at times ∼20 fs that is not present in the spectrum following HOMO ionization.

**FIG. 3.**
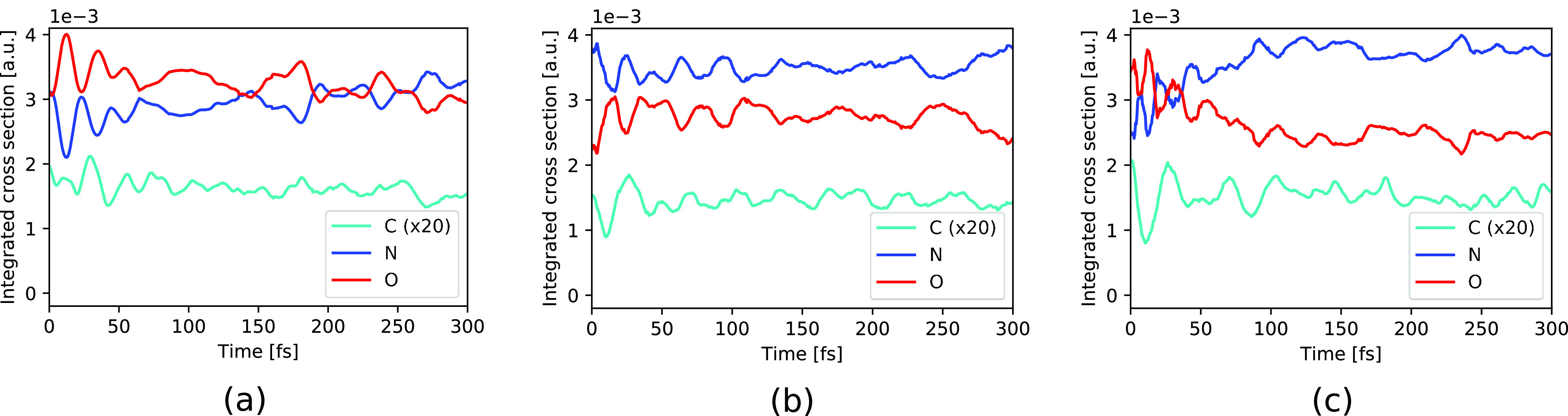
Energy-integrated spectra of urea from Fig. 2 for the C (scaled by 20), N, and O 1*s* pre-edge resonances following initial ionization of (a) HOMO, (b) HOMO-1, and (c) HOMO-2.

To investigate the intensity variation in more detail, Fig. 3 shows the absorption signal integrated over the energy range shown in Fig. 2. As seen for the initial HOMO and HOMO-1 holes [Figs. 3(a) and 3(b)], the energy-integrated spectra oscillate for the first 100 fs with a similar period as the oscillations seen in the energetic peak positions at the C and N *K*-edges for an initial HOMO hole state [Figs. 2(a) and 2(b)]. Apart from these oscillations, the energy-integrated spectra stay rather constant. In contrast, the absorption intensity at the N *K*-edge following ionization of HOMO-2 shows an overall increment in the first 100 fs, mirrored by a corresponding decrement in the absorption intensity at the O *K*-edge [Fig. 3(c)]. For all three cases, Fig. 3 indicates a strict anticorrelation between the temporal variations in the absorption intensities at the N and O *K*-edges.

**FIG. 4.**
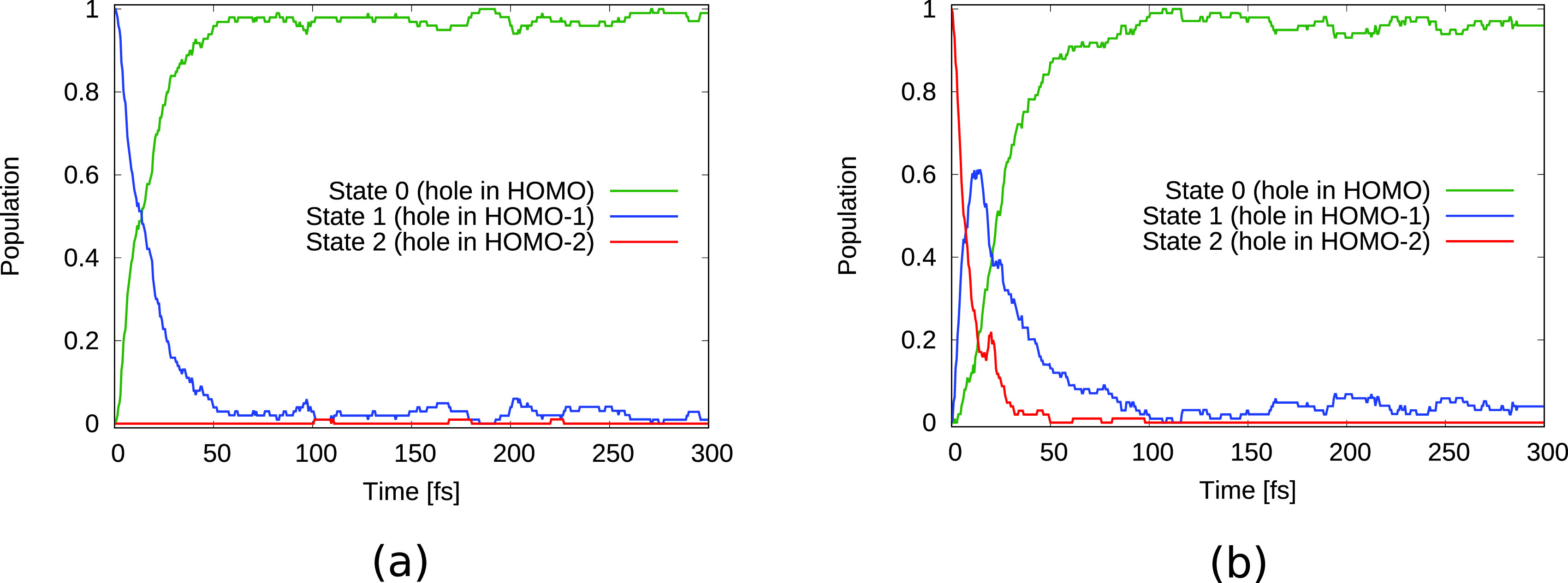
Electronic state population over time for urea with an initial hole in (a) HOMO-1 and (b) HOMO-2.

The observed temporal changes in the TRXAS for initial holes in HOMO-1 and HOMO-2 can be partially attributed to the electronic relaxation dynamics due to nonadiabatic effects. Figure 4 shows the population of the relevant electronic states as a function of time. As can be seen in Fig. 4(a), the HOMO-1 valence hole decays electronically on a timescale of ∼20 fs. The HOMO-2 hole state [Fig. 4(b)] decays rapidly toward the HOMO-1 hole state, losing half of its population within ∼5 fs, followed by a further decay leading to an almost complete relaxation to the HOMO-hole state by ∼100 fs. Since the HOMO-2 has dominant contributions on the O atom whereas the HOMO-1 is rather concentrated on the two N atoms [see Figs. 1(d) and 1(e)], the electronic relaxation step from the hole in HOMO-2 to the hole in HOMO-1 is reflected by the rapid decay of the O absorption signal and the corresponding increase in the N absorption signal [Fig. 3(c)]. Along with the reported electronic relaxations, the binding energy of the valence hole decreases and therefore the energetic gap to the core shells increases. The observed increase in the energetic position of the absorption resonances [Figs. 2(d)–2(i)] can thus be directly linked to these electronic relaxations.

#### 2. Identifying collective coordinates

**FIG. 5.**
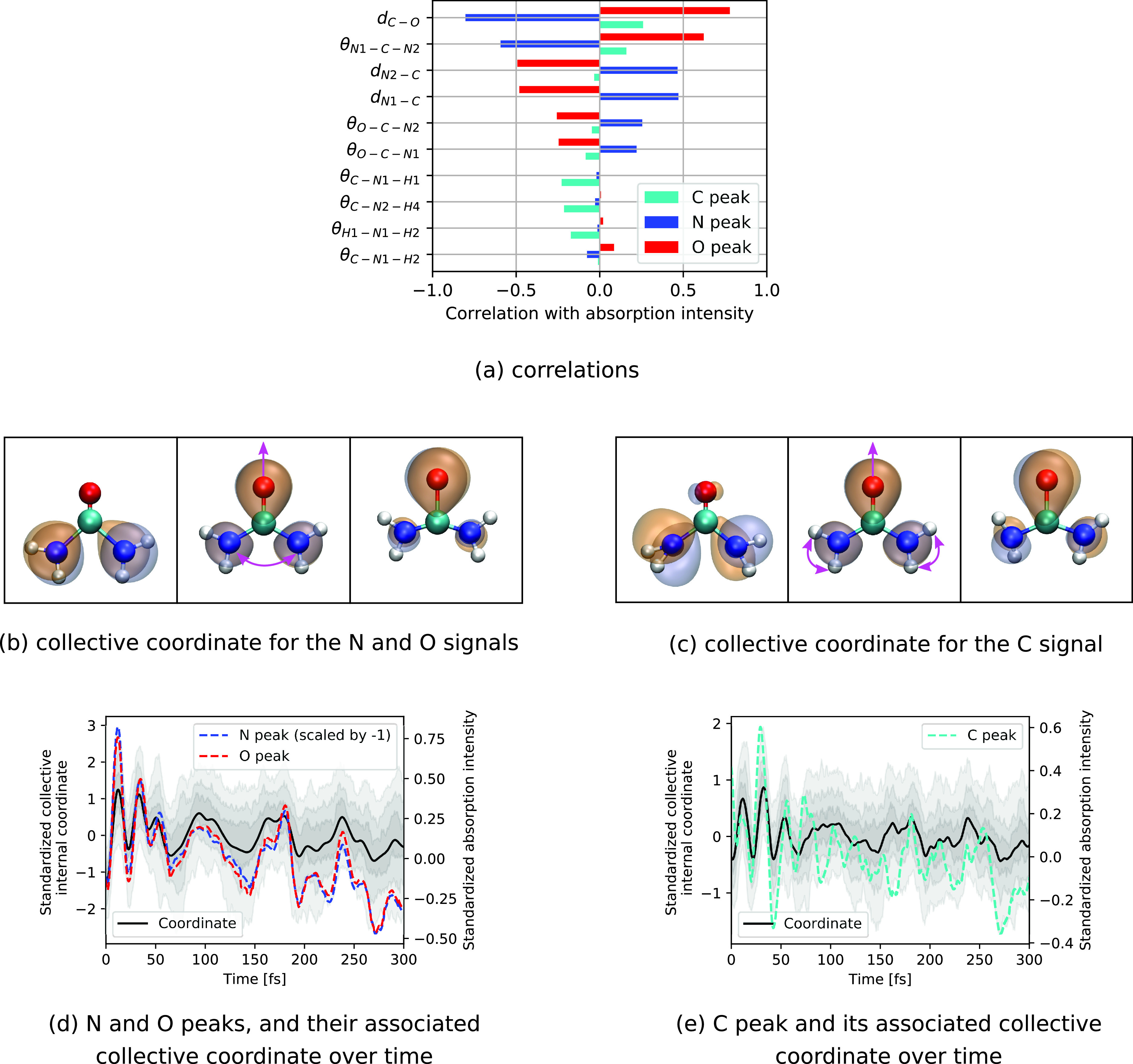
(a) Pearson correlation coefficients between internal coordinates of urea and absorption intensities at the C, N, and O *K*-edges for initial HOMO ionization. HOMO along the collective internal coordinate motion attributed to the temporal intensity variations (b) in the N and O peaks, and (c) in the C peak. Temporal evolution of the collective coordinate along with the averaged standardized absorption intensity for (d) the N and O peaks, and (e) the C peak. The shaded areas indicate regions between percentiles (37.5%–67.5%, 25%–75%, and 12.5%–87.5%) of the coordinate distributions.

Beyond interpreting the TRXAS via electronic relaxation processes, further insight can be obtained by understanding the spectral variations also in terms of structural changes. To that end, we have inspected the correlation of a complete set of internal coordinates with the three corresponding absorption intensities (note that the intensities from the two N atoms are summed up). We restrict this analysis to the trajectories with initial HOMO hole. However, the results can also be transferred to ionization of deeper valence orbitals, since the electronic states decay rapidly into the ground state of the cation. A similar analysis using the energetic positions of the absorption resonances instead of their intensities were employed before in Ref. 24, demonstrating how specific molecular vibrations can be linked to variations in the line positions. Figure 5(a) shows the Pearson correlation coefficients for the absorption intensities with the 10 internal coordinates showing the largest (anti)correlation. It can clearly be seen that the interatomic distances C-O, N1-C, and N2-C, and the angle N1-C1-N2 are the internal coordinates that show the strongest correlation or anticorrelation with the absorption intensities at the N and O *K*-edges. As already mentioned before [see Fig. 3(a)], there is a strong anticorrelation between the N and O signal intensities, which is reflected in the opposite sign of the correlation coefficients in Fig. 5(a). The absorption intensity at the C *K*-edge is rather correlated with the hydrogen angles C-N1-H1, C-N2-H4, and H1-N1-H2 as well as the interatomic distance C–O and the angle N1-C-N2.

To obtain a consistent picture of the correlations between absorption intensities and molecular vibrations, we have conducted a partial least squares regression analysis (PLSR)^43^ to find collective internal coordinates that maximize the covariance between the geometrical parameters and the absorption intensities. This way we have identified the two collective internal coordinates shown in Figs. 5(b) and 5(c), one linked to the absorption intensity at the N and O *K*-edges, and the other at the C *K*-edge. Accordingly, we can connect the variation in the N and O absorption signals to a collective coordinate predominantly consisting of C-O bond stretching and N1-C-N2 angle opening that explains 77% of the variance in the absorption intensities (which is the *R*^2^ score). We note that this motion represents only 10% of the total variation in the geometry, which is mostly dominated by hydrogen vibrations that do not significantly affect the intensities at the N and O *K*-edges. The impact of the obtained collective vibration on the absorption can be rationalized by the mechanism that stretching of the C–O bond causes the valence hole to be localized on the O atom. Hence, this leads to an increase in the absorption signal at the O *K*-edge and a decrease at the N *K*-edge [see Fig. 5(b)]. Similarly, we can attribute the variation in the C absorption signal to the collective motion illustrated in Fig. 5(c), which is mostly a correlated bending in the NH_2_ groups and to a lower extent an elongation of the C–O bond. This coordinate explains 30% of the variance in the absorption intensity and contributes 7% to the total variation in the geometry. As can be seen in Fig. 5(c), this bending motion deforms the HOMO, slightly shifting its contribution toward the C atom. The strong correlation of the respective time-dependent intensities with the two collective coordinates is illustrated in Figs. 5(d) and 5(e), which compare the evolution of the obtained coordinates with the change in their respective standardized absorption intensities.

### B. Urea dimer

**FIG. 6.**
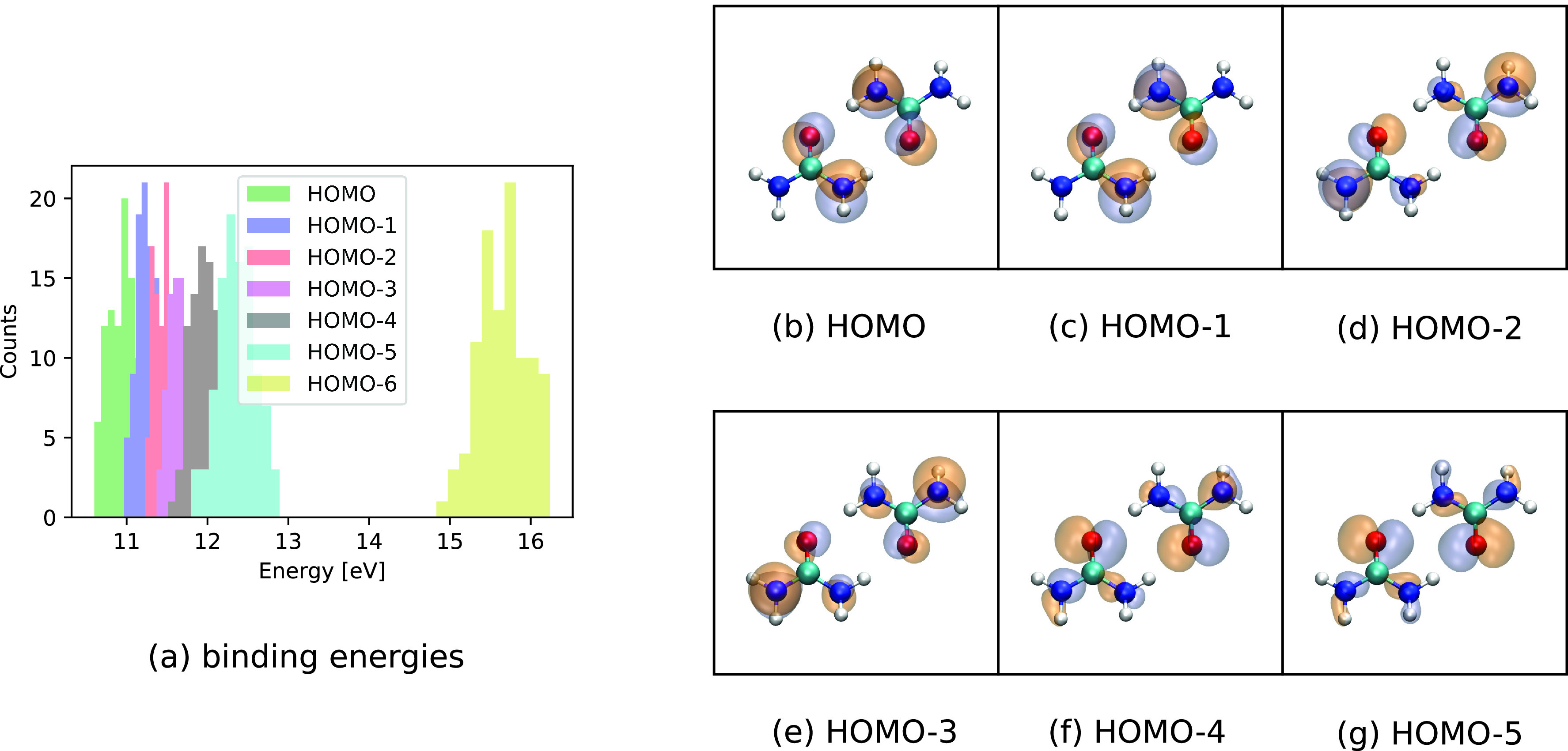
(a) Histogram of the calculated molecular orbital binding energies for HOMO to HOMO-6 for the ground-state Wigner distribution of neutral urea dimer. (b)–(g) HOMO to HOMO-5 at equilibrium geometry, visualized using VMD.^42^

Having understood how TRXAS features link to the ionization-induced dynamics of the urea monomer, we now turn to its dimer. Specifically, we have addressed here a cyclic urea dimer conformer, where the monomers exhibit two hydrogen bonds with each other leading to a more stable structure than the alternative linear conformers.^44^ Analogous to the monomer case, the binding energies of the orbitals up to HOMO-5 are relatively close in energy [Fig. 6(a)]. The molecular geometry of the considered conformer is depicted in Figs. 6(b)–6(g) together with the molecular orbitals HOMO to HOMO-5.

#### 1. TRXAS

**FIG. 7.**
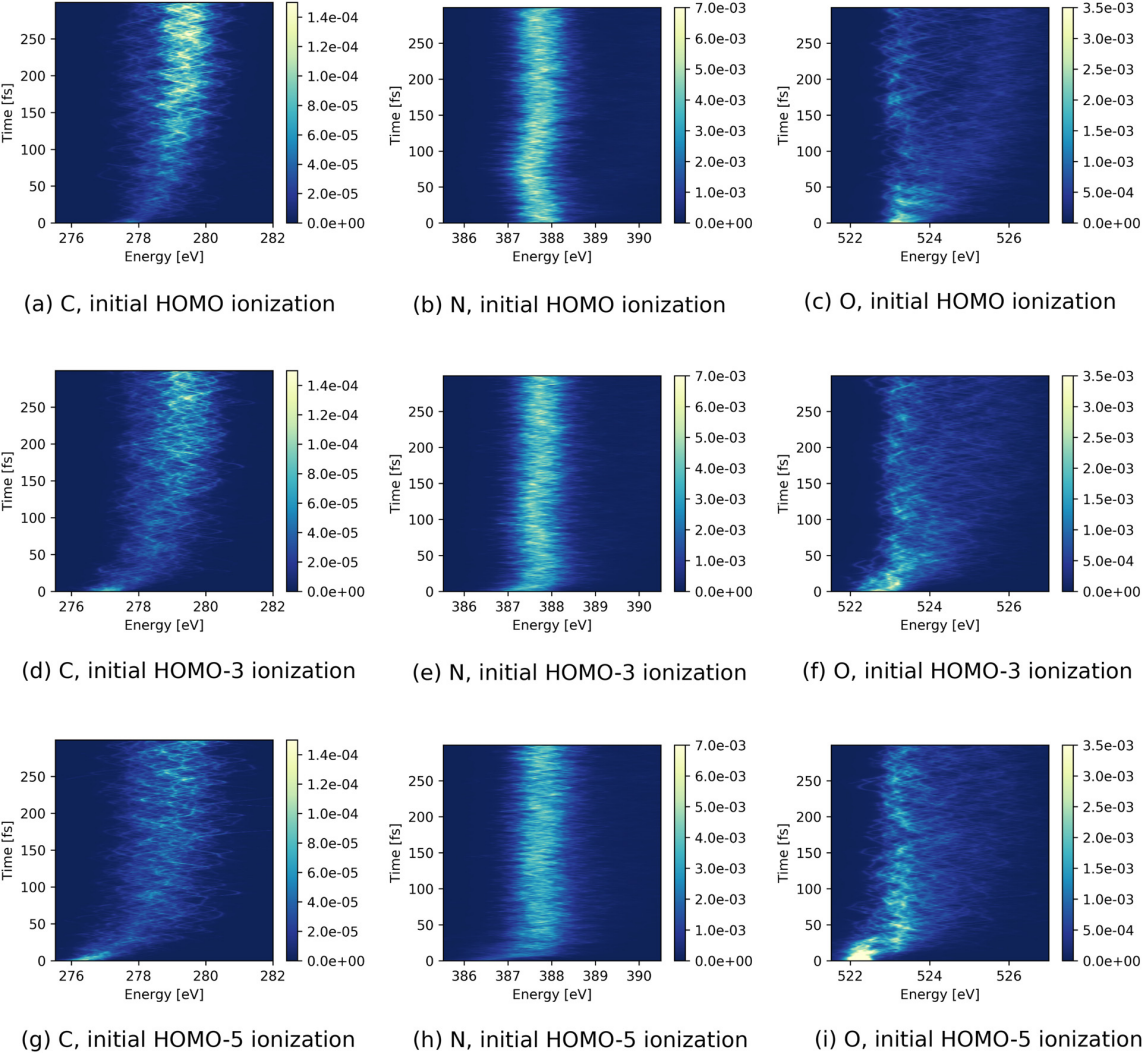
TRXAS (given by cross sections in a.u.) of urea dimer after initial ionization of (a)–(c) HOMO, (d)–(f) HOMO-3, and (g)–(i) HOMO-5 at the lowest (left) C, (middle) N, and (right) O *K*-edge absorption resonances.

Figure 7 shows the time evolution of the x-ray absorption spectra for various valence hole states in the ionized dimer. Exemplarily, we only show here the TRXAS for an initial hole in HOMO, HOMO-3, and HOMO-5. The spectra for HOMO-1, HOMO-2, and HOMO-4 show similar features to the ones shown in Fig. 7 (see the supplementary material). For all three initial valence holes, we see a pronounced change in absorption intensity at the C *K*-edge [see Figs. 7(a), 7(d), and 7(g)]. In particular, the very weak absorption signal becomes continuously stronger on a timescale of ∼100 fs. This increment is accompanied by a blueshift in the energetic position of the absorption line. In the absorption intensity at the O *K*-edge [Fig. 7(c)], there is a suppression of intensity occurring for the initial HOMO hole at ∼50 fs. For an initial hole in HOMO, a transient redshift in energy at ∼75 fs can be seen in the N absorption resonance position [Fig. 7(b)], which is absent for the deeper hole states.

As can be seen for the monomer, the TRXAS for the initial holes in HOMO-3 and HOMO-5 [Figs. 7(d)–7(i)] show spectral shifts in the absorption energy for all three edges at ∼20 fs and ∼40 fs for HOMO-3 and HOMO-5, respectively. For the HOMO-5 hole, the spectral shift is accompanied by a decline in the O absorption intensity [Fig. 7(i)].

**FIG. 8.**
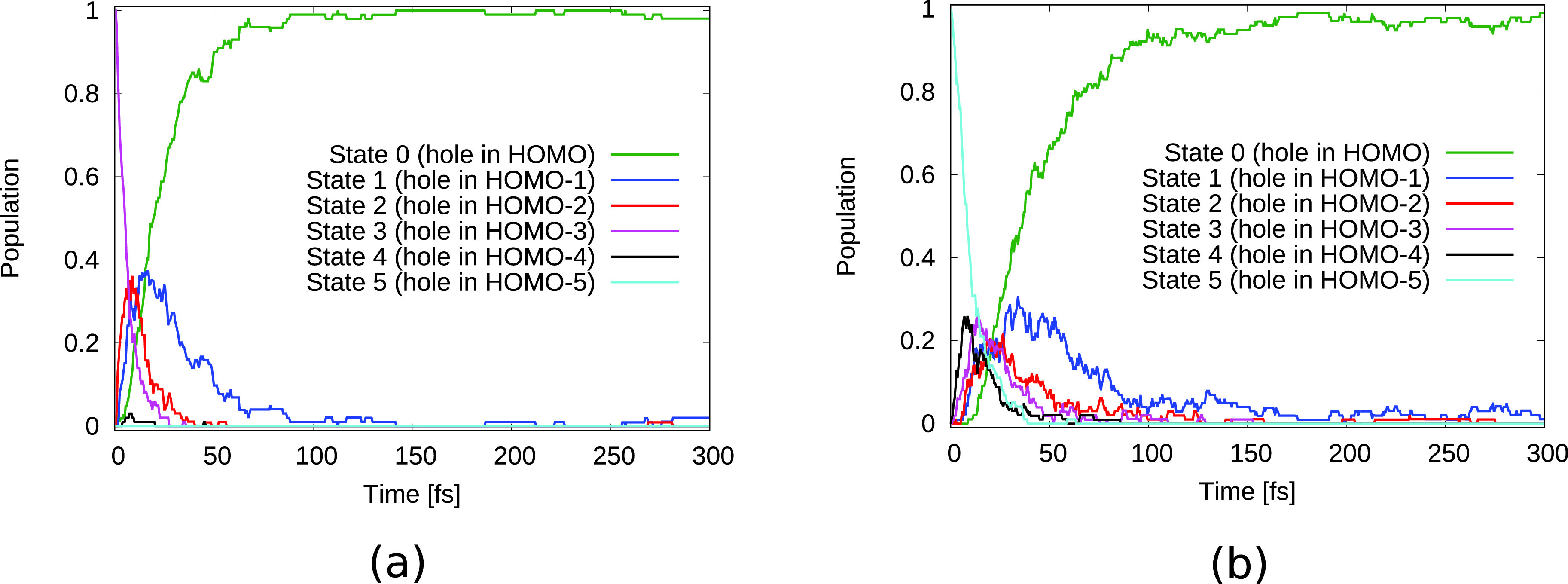
Electronic state population over time for urea dimer with an initial hole in (a) HOMO-3 and (b) HOMO-5.

The electronic state population dynamics for initial HOMO-3 and HOMO-5 holes are shown in Fig. 8. As can be seen for the two initial hole states, the electronic state rapidly relaxes to a state with a hole in HOMO-1 at ∼20 fs and ∼40 fs, respectively. This is followed by a further decay to the HOMO hole state at ∼40 fs and ∼60 fs, respectively. These timescales confirm the assignment of the spectral shifts in Figs. 7(d), 7(f), 7(g), and 7(i) to nonadiabatic relaxations. Around 100 fs after ionization almost all of the excited electronic state population has eventually relaxed to the ground state.

#### 2. Proton transfer reaction

Upon analyzing the trajectories, we have noticed that some of them exhibit a proton transfer reaction, where one of the protons forming a hydrogen bond moves toward the oxygen partner of the other monomer. This reaction has been observed in simulations with all the considered initial ionizations. However, the proton transfer only occurs after relaxation to the HOMO ionized state. To quantify this process, we have employed a criterion for proton transfer to be defined by the distance between the oxygen on the acceptor urea and the hydrogen from the donor being less than 1.25 Å. Using this criterion, we have found that after ionization of HOMO 70% of the trajectories undergo proton transfer on a timescale of ∼50 fs (estimated by fitting a sigmoid function to the number of completed proton transfers over time). For initial ionization in HOMO-1, HOMO-2, HOMO-3, HOMO-4, and HOMO-5, we have observed timescales of about 65 fs, 80 fs, 80 fs, 90 fs, and 95 fs, and a relative proportion of the trajectories undergoing the transfer of 60%, 53%, 54%, 55%, and 40%, respectively. We attribute this slightly delayed proton transfer for initially deeper bound valence hole states to the additional time that is needed to relax to the ground state where the proton transfer eventually occurs.

**FIG. 9.**
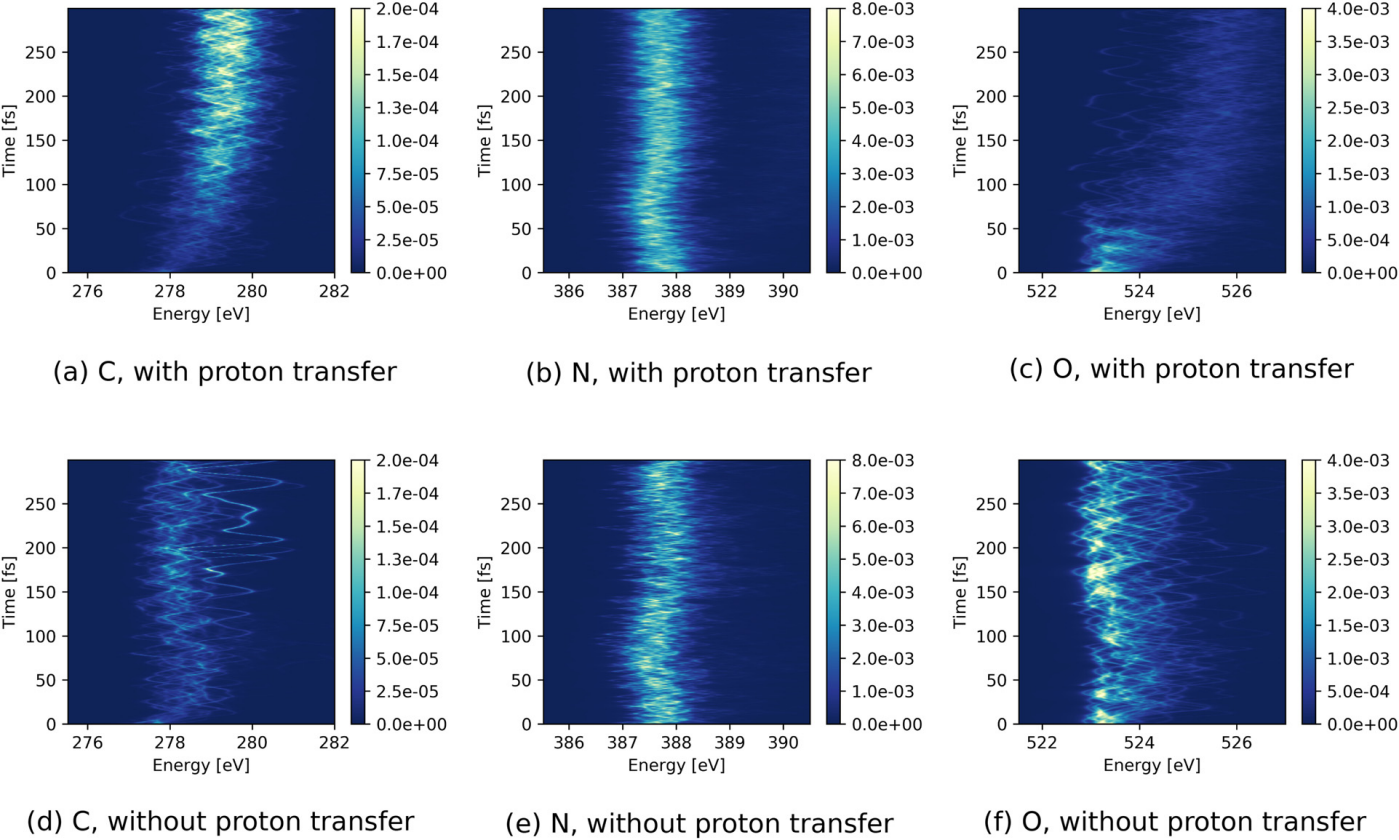
TRXAS (given by cross sections in a.u.) of urea dimer after initial ionization of HOMO for trajectories (a)–(c) with and (d)–(f) without proton transfer at (left) C, (middle) N, and (right) O *K*-edge absorption resonances.

To investigate the effect of proton transfer on the spectra, we have split the trajectories up into two subsets, one that undergoes proton transfer and another that does not, based on the criterion stated above. The respective spectra for the two subsets are shown in Fig. 9. Since the proton transfer occurs in the HOMO hole state, we have restricted this analysis to trajectories starting in this hole state. Both subsets show very similar dynamical features for the N *K*-edge where a transient shift toward lower energies is seen at ∼75 fs [Figs. 9(b) and 9(e)]. For absorption resonance at the C and O *K*-edges, the two subsets exhibit distinct features for delay times larger than 50 fs. The trajectories undergoing proton transfer show a considerable blueshift in energetic position at the C *K*-edge absorption resonance and a rise in intensity after ∼100 fs [Fig. 9(a)]. In contrast, only a relatively minute rise in intensity [Fig. 9(d)] and no energetic shifts can be seen for the subset without proton transfer. Furthermore, for the subset with proton transfer, the absorption intensity at the O *K*-edge declines at ∼50 fs [Fig. 9(c)], whereas for the subset without proton transfer no systematic change in intensity can be seen [Fig. 9(f)].

**FIG. 10.**
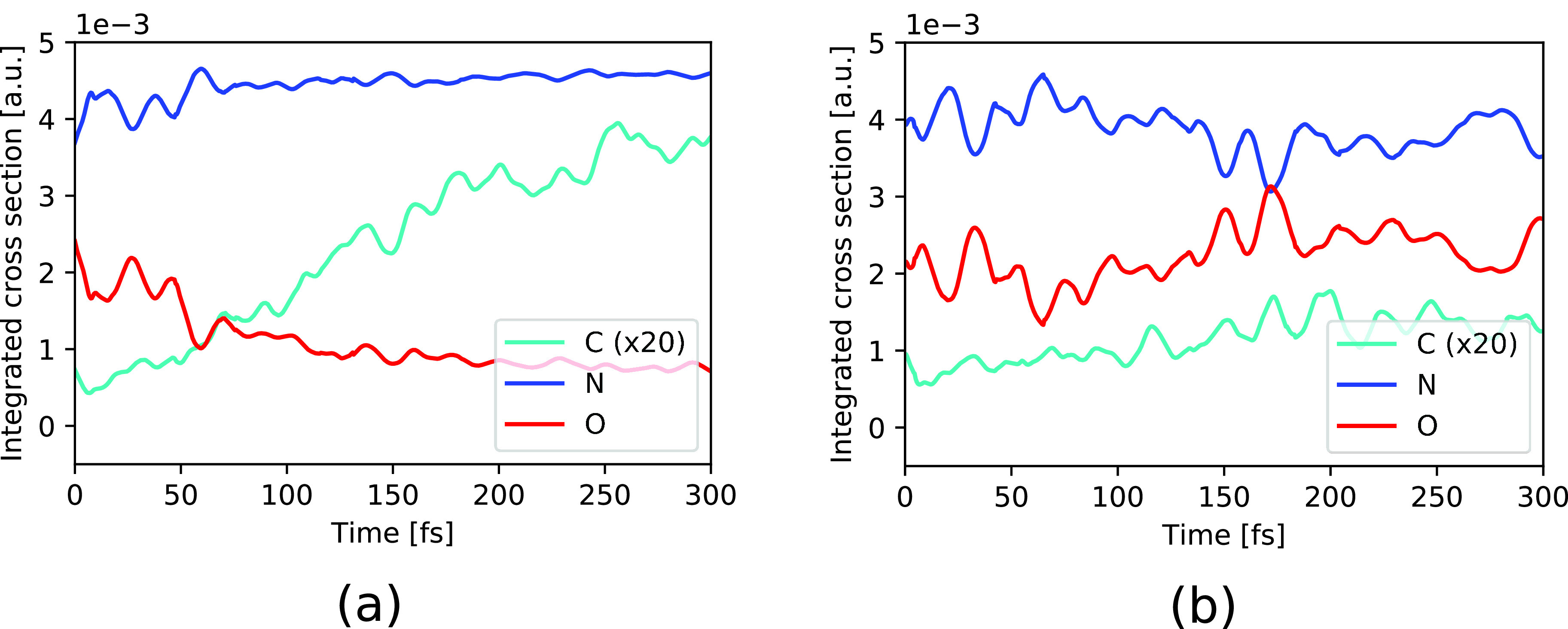
Energy-integrated spectra of urea dimer from Fig. 9 for the C (scaled by 20), N, and O 1*s* pre-edge resonances following initial ionization in HOMO for trajectories (a) with and (b) without proton transfer.

The reported changes in absorption intensities can be more clearly seen in the energy-integrated spectra in Fig. 10 for the two cases with and without proton transfer. The continuous rise in intensity at the C *K*-edge well beyond 100 fs and the decrease in signal at the O *K*-edge up to ∼50 fs for trajectories with proton transfer are clearly visible in Fig. 10(a). Moreover, for the trajectories with proton transfer, one can see that for times less than 50 fs the decrease in the O intensity is mirrored by an increase in the N intensity. For trajectories without proton transfer in Fig. 10(b), the intensities exhibit oscillations but no clear increment or decrement. Similar to the monomer case, we see here a strong anticorrelation for the absorption at the O and N *K*-edges, suggesting that the time variation can be linked to the same structural dynamics.

**FIG. 11.**
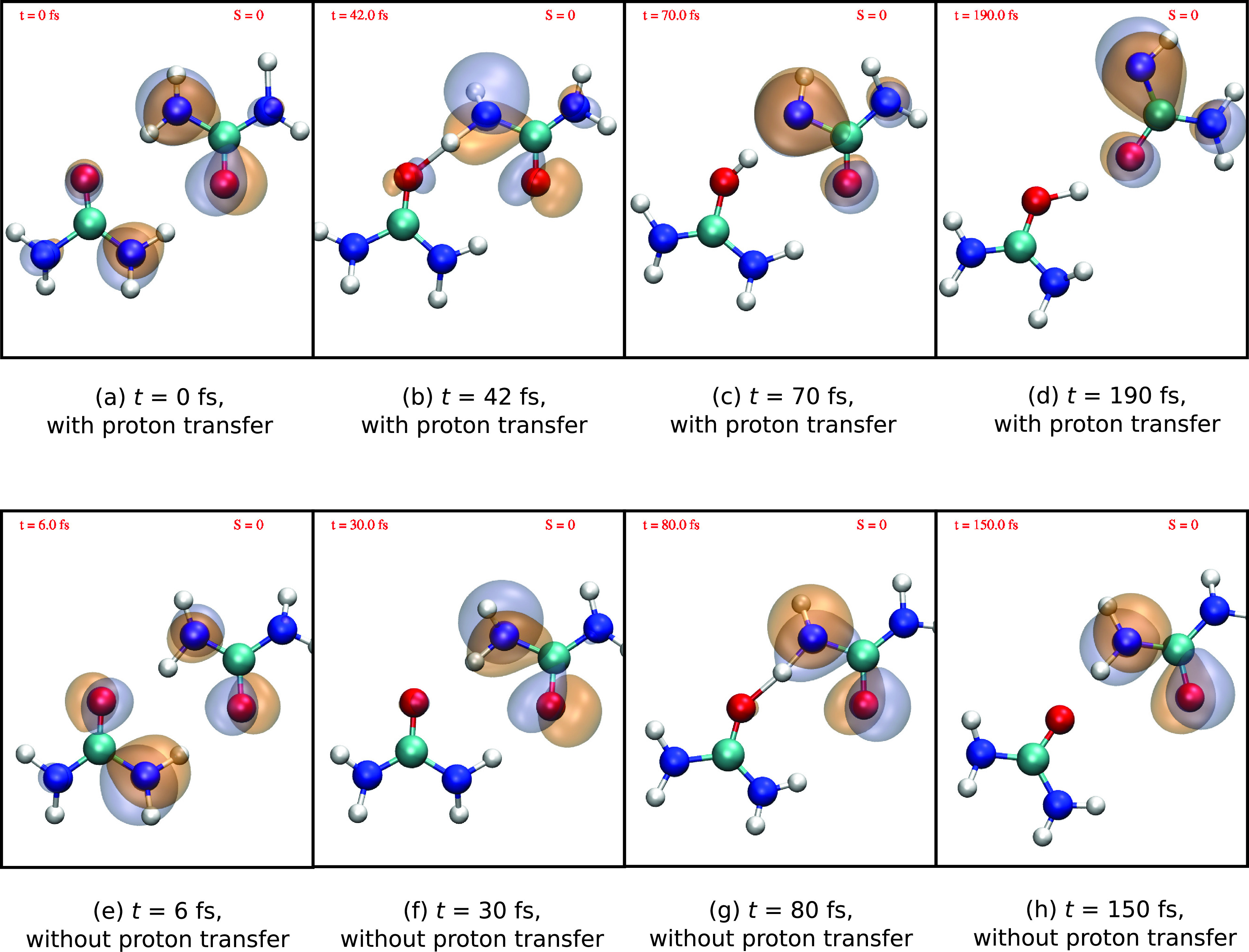
Hole orbital at selected times for two exemplarily trajectories, (a)–(d) one with and (e)–(h) another without proton transfer following initial HOMO ionization. The hole remains in HOMO (labelled by S = 0) throughout the simulation.

To understand these intensity variations, Fig. 11 shows snapshots at selected times for two representative trajectories, one with proton transfer [Figs. 11(a)–11(d)] and another without [Figs. 11(e)–11(h)]. The HOMO orbital containing the electron hole is depicted in each of the snapshots. In both trajectories, the initially delocalized valence hole localizes to one of the two ureas after ∼30 fs [see Figs. 11(a), 11(b), 11(e), and 11(f)]. The initial electron hole localization is followed by cleavage of one hydrogen bond and a contraction of the other hydrogen bond. The upper trajectory exhibits proton transfer leading to a protonated urea cation (NH_2_)_2_COH^+^ and a deprotonated urea (NH_2_)(NH)CO [Fig. 11(c)]. After the proton transfer, the dimer rearranges and a new hydrogen bond is formed between the O atoms of (NH_2_)_2_COH^+^ and (NH_2_)(NH)CO [Fig. 11(d)]. In contrast, in the trajectory without proton transfer, the dimer remains bonded via one remaining hydrogen bond between the N and O atoms [Fig. 11(h)]. In the corresponding snapshots in Figs. 11(c) and 11(d), one can see that the proton transfer comes along with a deformation of the HOMO orbital leading to larger and lower contributions on the N and O atoms, respectively. From the inspection of these example trajectories, we thus hypothesize that the observed decrease in O absorption intensity and the accompanying increase in the N absorption intensity [Fig. 10(a)] can be attributed to this reaction.

#### 3. Identifying collective coordinates

**FIG. 12.**
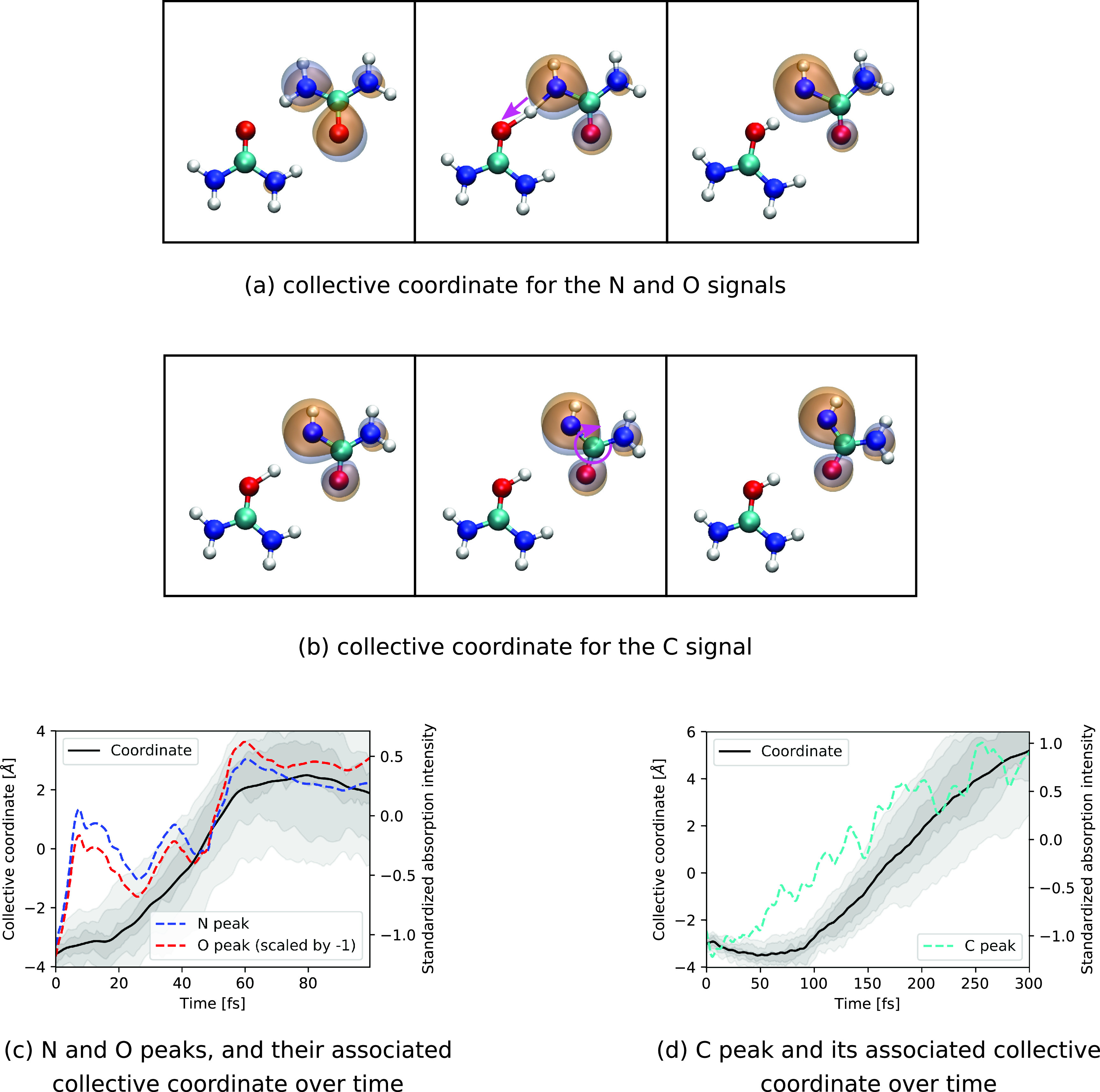
HOMO along the collective coordinate motion that explains the intensity variations (a) in the N and O peaks, and (b) in the C peak for the dimer. Temporal evolution of the collective coordinate along with the averaged standardized absorption intensity for (c) the N and O peaks, and (d) the C peak. The shaded areas indicate regions between percentiles (37.5%–67.5%, 25%–75%, and 12.5%–87.5%) of the coordinate distribution.

To verify our interpretation of the intensity variations with the dynamical motifs of the proton transfer reaction, we have performed a PLSR analysis on the geometrical and spectral variations. Due to the difficulties in properly selecting a complete set of internal coordinates that also describe intermolecular motion such as the proton transfer, the analysis has been conducted using Cartesian coordinates as opposed to the monomer where internal coordinates have been used. Due to the symmetry, any of the two hydrogen bonds between the two ureas can exhibit proton transfer. To get a clearer picture, we have selected trajectories undergoing proton transfer only in one particular direction as input for the PLSR. Because the intensity variation for the N and O signal occurs in the first 100 fs of the simulation, we have restricted the corresponding analysis to this time range. The collective coordinate obtained for the N and O signals is shown in Fig. 12(a). As one can see, the resulting coordinate describes the proton transfer and comes along with a shift in the HOMO from the O toward the N atoms. This result confirms our interpretation that the decrease in the absorption signal for O and the corresponding increase for N is indeed a signature of the proton transfer. Figure 12(c) compares the time evolution of this collective motion with the standardized N and O absorption intensity. As can be seen, considerable variations in the intensity in the first 20 fs cannot be explained with the depicted motion (the coordinate explains 24% of the variation in the N and O peaks combined). This discrepancy indicates that the relation between geometrical changes and absorption intensity cannot fully be explained with a one-dimensional model. Nevertheless, on a longer timescale, the depicted coordinate and the absorption intensity follow the same trend.

To also obtain an explanation for the increment in absorption signal at the C *K*-edge seen at ∼100 fs [Fig. 9(a)], we have performed a similar PLSR analysis on the absorption intensities (again taking only trajectories with proton transfer in one direction), but now with the full time range up to 300 fs. We find the collective coordinate shown in Fig. 12(b) that is able to describe 45% of the variance in the C absorption intensity. The coordinate depicts the rearrangement of the donor urea after the proton transfer, where (NH_2_)(NH)CO rotates and forms a new hydrogen bond with (NH_2_)_2_COH^+^ between the two O atoms. As can be seen, the formation of the new hydrogen bond between the two O atoms results in an increase in the population of the HOMO orbital on the C atom [also seen in Figs. 11(c) and 11(d)] and thus explains the increase in absorption intensity at the C *K*-edge. The evolution of the identified coordinate together with the averaged variation in the standardized C absorption intensity is shown in Fig. 12(d). As can be seen, the evolution of this coordinate follows roughly the intensity rise in the C resonance. However, in the range below 100 fs, the rise in intensity does not follow the evolution of the collective coordinate. A further analysis focusing on the time-range up to 100 fs (see the supplementary material) reveals that this rise can be associated with a proton transfer motion similar to the one depicted in Fig. 12(a).

To sum up, we have observed that the localization of the electron hole to one of the ureas causes a redistribution of the charge in the dimer and alters the partial charge distribution between the hydrogen bond partners. This leads to a repulsion along one of the hydrogen bonds and to attraction along the other hydrogen bond. After the proton has been transferred to the acceptor molecule, the acceptor molecule is now positively charged and attracts the partially negative O atom of the now neutral deprotonated urea. This causes the formation of a new hydrogen bond between the O of the donor with the donated proton on the acceptor side. The proton transfer as well as the subsequent rearrangement of the resulting conformer leave distinct fingerprints as intensity variations in the TRXAS.

Regarding the proton transfer, we note that an analogous sequence of events was described before in a study of liquid water, where the localization of a valence hole triggered a proton transfer and thus separation of charge and spin.^45^ In this context, an x-ray absorption energy shift for the donor during proton transfer was reported,^15^ similar to what has been observed here as the transient redshift at ∼75 fs in the energetic position of the N resonance [Figs. 9(b) and 9(e)]. To further investigate this transient shift, we have performed a similar PLSR analysis as for the N and O absorption intensities, but now for the energetic position of the N absorption resonance using all trajectories, with and without proton transfer, taking the first 100 fs. The results confirm that this transient energy shift can be attributed to the motion of attempted proton transfer which all trajectories undergo (see the supplementary material). More specifically, this is the breaking of one hydrogen bond and contraction of the other. The analogies to the case with liquid water lead us to speculate that the discussed trends in the TRXAS are transferable to other situations where a proton transfer takes place. Notably, further analysis (not shown) reveals that these attempted proton transfer dynamics can also partially account for the discrepancy in the first 20 fs in Fig. 12(c).

## IV. CONCLUSION

To summarize, we have performed on-the-fly *ab initio* nonadiabatic dynamics upon HOMO and deeper valence ionization of urea and its dimer and have calculated the TRXAS at the C, N, and O *K*-edges. Nuclear as well as electronic dynamics are clearly visible as distinct spectroscopic signatures in the TRXAS. After ionization in deeper valence orbitals, time-dependent blueshifts in the absorption energy are observed, indicating electronic relaxation via nonadiabatic transitions to the electronic ground state of the cation. This occurs on a timescale of a few tens of femtoseconds in both urea and its dimer. Creating an initial hole in HOMO for urea leads to some noticeable variations in the intensity of the spectra that we associate with specific molecular vibrations. For the urea dimer, a proton transfer reaction occurs between the two ureas following HOMO ionization at around 50 fs for about 70% of the trajectories. This reaction leaves fingerprints in the TRXAS which can be attributed to the proton transfer itself and a subsequent rearrangement of the dimer structure.

Given the technical advances in HHG and XFEL light sources, experiments with increasingly better temporal and spectral resolution are now in reach. This has enabled TRXAS to become a reliable tool for inspecting ultrafast chemical dynamics.^10–13,15–18^ However, the interpretation of TRXAS spectra still crucially relies on insights from simulations. By providing a detailed description of how the dynamics of a simple, yet biologically relevant, molecule is reflected in the TRXAS, we have demonstrated in this work how statistical analysis tools can be used to unravel the often complicated relationship between these two features. This step becomes particularly crucial when large molecules are addressed, where the numerous vibrational degrees of freedom prohibit a clear interpretation via visual inspection of the simulated trajectories. We hope that the simulation and analysis methods used here will inspire future work on gaining further insights into the effects of ionizing radiation on bigger biological systems.

## SUPPLEMENTARY MATERIAL

See the supplementary material for the TRXAS of urea dimer for an initial hole in HOMO-1, HOMO-2, and HOMO-4, and the figure illustrating the collective coordinate for the N absorption energy of the dimer.

## Data Availability

The data that support the findings of this study are available from the corresponding authors upon reasonable request.
